# Sensitivity of the iFalcon^TM^V700 rebound tonometer to measurement distance, angle and position in ex-vivo rabbit eyes

**DOI:** 10.1186/s12917-026-05747-7

**Published:** 2026-07-20

**Authors:** Jing Jin, Hao Cheng, Yunhao Su, Ji Fu

**Affiliations:** The School of Mechanical Engineering, Zhejiang Sci-Tech University, Hangzhou, China

**Keywords:** Intraocular Pressure, Rebound Tonometry, Ex-Vivo, iFalcon^TM^V700, New Zealand White Rabbit

## Abstract

Rebound tonometers are widely used for intraocular pressure (IOP) monitoring in animal experiments. However, previous studies and clinical experience have demonstrated that their measurements are susceptible to operator-dependent factors, such as variations in measuring distance, probe angle, and corneal impact position, all of which may contribute to reading variability. To evaluate the measurement stability of the iFalcon^TM^V700 rebound tonometer under different measuring conditions, this study employed a stepwise pressure-increasing protocol to set eight target IOP levels (10–50 mmHg) in two enucleated rabbit eyes (*n* = 2). We systematically investigated the effects of measuring distance (4–7 mm), measuring angle (approximately 30° superior, inferior, left, and right relative to the corneal normal), and corneal measuring location (central cornea and 3 mm peripheral cornea) on IOP readings. Paired t-tests were used to compare measurements between each test condition and its corresponding reference condition. Stratified analyses were performed across different IOP levels to assess the potential influence of IOP. The results showed that the maximum mean differences caused by distance, angle, and position factors were − 1.8125 mmHg, − 0.9450 mmHg, and − 1.8175 mmHg, respectively, all of which were less than 2 mmHg, with low measurement dispersion at each IOP gradient. Although the iFalcon^TM^V700 exhibited statistically significant dependent biases under the aforementioned operating conditions, the absolute magnitudes were limited and had little impact on experimental interpretation and conclusions, suggesting that the device demonstrates reasonable measurement stability under the tested conditions.

## Introduction

IOP refers to the pressure exerted by intraocular contents on the ocular wall, and its accurate measurement forms the foundation for clinical diagnosis and treatment, disease screening, and basic experimental research in ophthalmology [1]. Glaucoma represents the leading cause of irreversible blindness worldwide, with elevated IOP constituting its most significant modifiable risk factor [2, 3]. Precise IOP measurement is not only critical for the early diagnosis of glaucoma but also serves as an important indicator for assessing treatment efficacy and determining prognosis [4]. Accurate IOP quantification is equally important in veterinary ophthalmology and in animal experiments, particularly those using rabbit eyes, providing core datasets for studies on ocular pathological mechanisms and the development of novel therapeutic agents [5, 6]. Notably, their corneal biomechanical properties and anterior chamber anatomy closely resemble those of human eyes [7, 8], while their moderate corneal diameter and curvature enable precise and repeatable manipulation of probe-cornea distance, measurement angle, and impact position [9], rendering them an ideal model for systematically evaluating operator-dependent factors.

Commonly used tonometers include applanation, indentation, and rebound devices. Among these, rebound tonometry has been widely applied in clinical and experimental research owing to its ease of operation, lack of need for topical anesthesia, and suitability for various animal models. Its working principle is based on the deceleration and rebound dynamics of a lightweight probe after impacting the cornea. A built-in algorithm analyzes the probe’s motion parameters and converts them into IOP values [10–12]. Although rebound tonometry has many advantages, its measurement accuracy is susceptible to operational factors, representing a key bottleneck that limits its measurement stability [13, 14]. Existing studies have shown that measurement distance, probe angle deviation, and corneal impact position can all cause significant fluctuations in IOP readings.

Regarding measurement distance, evidence indicates that the rebound tonometer has a certain tolerance for chicken eyes within a distance range of 3–5 mm [15]. However, it remains unclear whether this distance window applies to other animal models, such as rabbits. The extent of deviation beyond this window also requires systematic quantification. Recent studies further show that in canine and rat eyes, probe-cornea distance has a more pronounced effect on readings. IOP readings at distances of 4, 6, and 8 mm differ significantly (*P* < 0.01), and the measured values increase with distance [16]. These findings suggest that the distance effect is distinctly species-dependent, and its magnitude varies across species. Notably, however, systematic data regarding the distance tolerance of rebound tonometers in rabbit eyes remain scarce, and it remains unclear whether findings derived from chicken, canine, and rat models can be extrapolated to rabbits. This knowledge gap prevents the establishment of evidence-based guidelines for distance adjustment in rabbit IOP measurements.

Regarding measurement angle, existing conclusions remain divergent. Chicken eye studies found poor tolerance to angular deviation within the 0°-20° off-axis range (*P* < 0.05) [15]; canine eye studies showed that tilted or peripheral measurements caused mild underestimation, with differences ranging from 0 to 2.9 mmHg compared to central perpendicular measurements [17]; in rabbit eyes, no significant change in measured values was observed within a 20° probe tilt angle [18]; whereas in human eyes, a 10° or 20° probe tilt caused readings to be significantly lower than those obtained with Goldmann applanation tonometry. The 95% limits of agreement (LoA) between the two devices under different conditions ranged from − 5.6 to − 4.5 mmHg [19]. These discrepancies suggest that the angle tolerance threshold is distinctly species- and device-specific. Nevertheless, available data evidence in rabbit eyes is limited to a single device and a single angular condition, leaving the angular tolerance of newer devices—particularly those incorporating nonlinear algorithms—largely unexplored. Given the growing variety of rebound tonometers available for animal research, the operational robustness of each device‑species combination requires independent validation to ensure reliable interpretation of readings.

Regarding measurement position, current evidence indicates that peripheral corneal measurements yield lower IOP values than central corneal measurements. Human eye studies have confirmed that central IOP is significantly higher than peripheral IOP (by approximately 3–4 mmHg). Peripheral measurements can underestimate by about 1.4-2 mmHg [20]. Further studies suggest that this positional effect primarily arises from differences in corneal curvature and probe impact angle, rather than corneal thickness itself [21]. Eccentric probe positioning can also lead to either overestimation or underestimation of IOP. This indicates that precise alignment between the probe and the cornea is critical for stable measurements [22]. In summary, rebound tonometry is widely used in clinical and experimental research. However, its measurement accuracy is influenced by operating conditions, including distance, angle, and position. Existing evidence also shows inconsistencies across different species and devices. Such limitations indicate that the operational robustness of a specific device in a target animal model requires systematic validation. This highlights the necessity of systematic evaluation of the iFalcon^TM^V700 to establish its operational tolerance in the rabbit model.

To address these limitations, the iFalcon^TM^V700 incorporates a nonlinear physical model based on Hertzian contact theory [23]. Theoretically, the nonlinear model analyzes the probe’s dynamic attenuation curve after corneal impact. This may allow for more accurate IOP estimation, especially for species with specific corneal biomechanical properties, such as rabbits. It may also reduce measurement deviations caused by varying operational conditions. Recent ex vivo rabbit study utilizing the predecessor model iFalcon^TM^V100 confirmed the accuracy of this approach. The iFalcon^TM^V100 readings showed a strong linear correlation with implantable pressure sensor readings (r² = 0.97), with a mean bias of -0.39 mmHg [24]. The iFalcon^TM^V700 maintains the identical core algorithm while adding support for additional species. Accordingly, given the shared core algorithm, the iFalcon^TM^V700 is expected to provide favorable accuracy under ideal conditions. However, the aforementioned study did not address the influences of operational factors such as measuring distance, angle, and position. In practice, small variations in operational conditions in practical applications may increase the dispersion of experimental data and weaken the reliability of research conclusions. Therefore, systematic investigation is needed to understand how operational factors such as distance, angle, and position affect the measurement stability of the iFalcon^TM^V700. Clarifying its operational robustness will promote its standardized application.

The aim of this study was to systematically evaluate the effects of measurement distance, angle, and corneal position on the measurement stability of the iFalcon^TM^V700 rebound tonometer in ex vivo rabbit eyes. Accordingly, this study used ex vivo rabbit eyes as a model. Stepwise pressure-increasing protocol was adopted to establish 8 distinct IOP levels (10–50 mmHg). We examined three operational factors: measuring distance (4–7 mm), measuring angle (0° and four directional deviations), and corneal measuring position (central and 3 mm peripheral cornea). A measuring distance of 4 mm, middle angle, and central corneal position were used as reference conditions. For each target IOP level, the mean, standard deviation, and mean bias of measurements were calculated under different operating conditions. This allowed us to evaluate the device’s measurement stability and operational robustness across varying IOP levels. These findings provide an experimental reference for the standardized application of this device in clinical practice and experimental research.

## Materials and methods

### Participants

Two healthy rabbit eyes were used in this study. They were obtained from two adult New Zealand white rabbits (one eye per rabbit) and purchased from a local animal experiment center (Lifutai Bio Co., Ltd.). Given that the two eyes from the same individual have similar corneal biomechanical properties and IOP measurement performance, including both eyes could introduce pseudoreplication. This may compromise the validity of statistical inference. Accordingly, only one eye per rabbit was selected in this study [25, 26]. 

All eyes were obtained postmortem. The rabbits had been euthanized as part of other independent research projects. No animals were sacrificed specifically for this study, and no live animals were used. Only eyes with no known history of ocular disease or intervention were selected post-euthanasia. All procedures were performed by a qualified veterinarian. First, overdose anesthesia was induced by intravenous injection of propofol (10 mg/kg) via the marginal ear vein. Immediately after anesthetic induction, euthanasia was performed by exsanguination via carotid artery. Bilateral enucleation was performed, but only one eye from each rabbit was used in this study. The eyes were rinsed immediately with sterile normal saline after enucleation. Subsequently, a board-certified veterinary ophthalmologist examined all eyes via slit-lamp biomicroscopy to confirm gross corneal clarity, anterior chamber depth, and lens position. No macroscopic structural abnormalities were observed, such as corneal scarring, synechiae, and significant opacities. The enucleated globes were placed in a sterile sealed container filled with sterile normal saline, stored on ice, and transported to the laboratory after dissection. During the subsequent experiments, the corneal surfaces were kept moist by regular application of sterile normal saline using a dropper. All experiments were completed within 12 h post-enucleation. Notably, this examination was designed to exclude major defects without fluorescein staining. Therefore, subtle surface irregularities, such as superficial corneal ulcers, could not be ruled out. The potential impact of this limitation is addressed in the Discussion.

Two ex vivo rabbit eyes were used in this study. Each eye was assigned to four target IOP levels, covering eight pressure gradients (10–50 mmHg). Six repeated measurements were taken for each measuring condition (distance, angle, position) at each pressure point. This yielded a total of 672 data points. This design partially compensated for the limited sample size by increasing the number of measurements and condition gradients, and followed the common practice of using a small sample for proof-of-concept validation in ex vivo ophthalmic device studies [27, 28]. Nevertheless, we acknowledge that a sample size of two eyes inherently limits the generalizability of our findings. With only two biological replicates, we cannot account for interindividual variability in corneal biomechanical properties, such as central corneal thickness, hydration status, or age-related differences, all factors that may affect tonometric performance. Therefore, our results should be interpreted as an initial proof-of-concept characterization of the device’s operational tolerance under controlled ex vivo conditions, rather than a definitive assessment of its clinical performance. The impact of this limited sample size on the generalizability of the results has been addressed as a key limitation in the Discussion section.

### IOP measurement

**Fig. 1:**
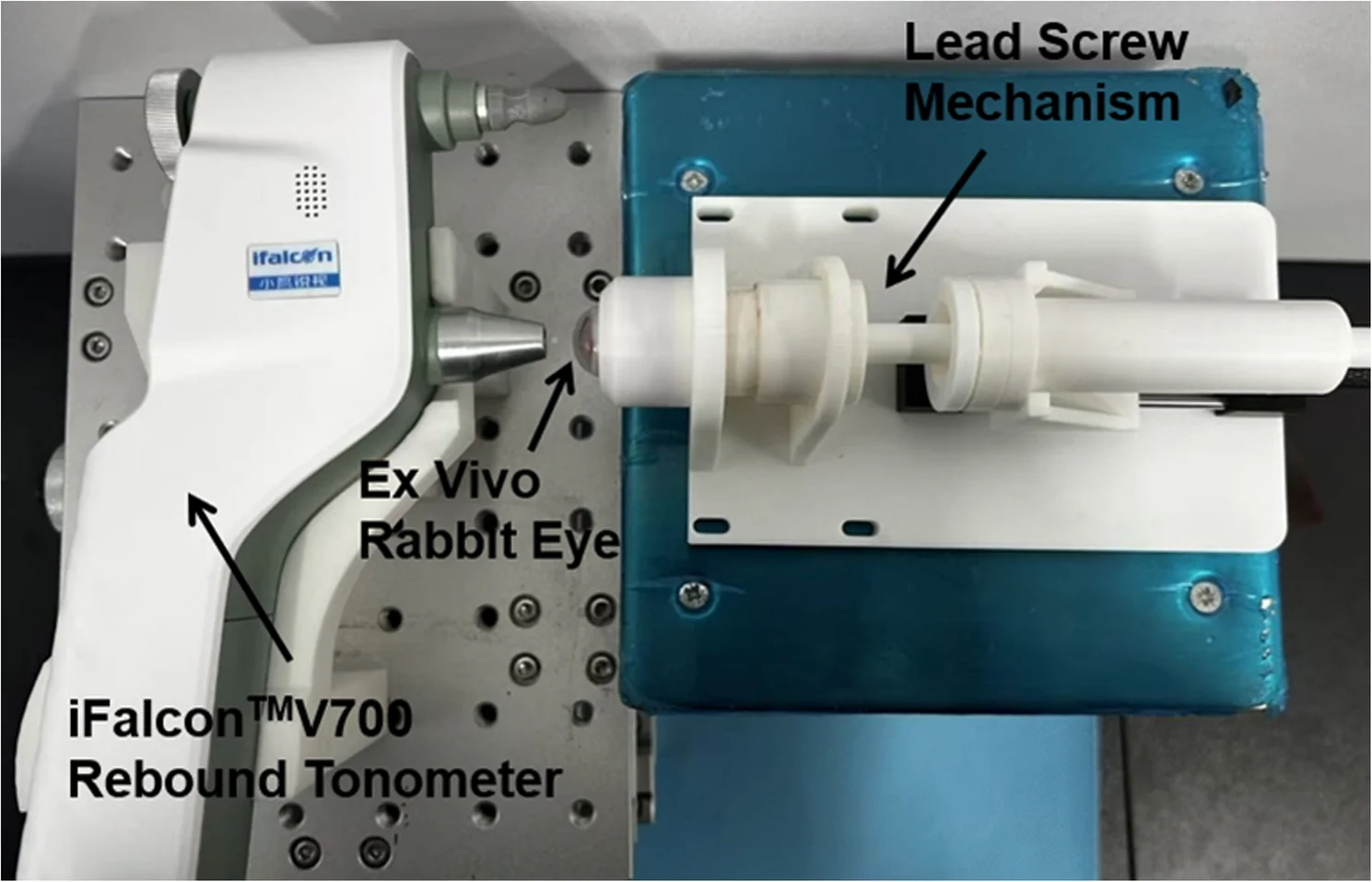
Experimental platform setup

Customized three-dimensional (3D) compression device (Fig. 1) was used to pressurize the rabbit eyes. The core of the device consists of a lead screw transmission mechanism: rotating the lead screw drives the movable plate in contact with the rabbit eye to shift horizontally, thereby achieving controlled adjustment of IOP. The device was not independently validated against a reference pressure sensor; therefore, the reported IOP levels represent nominal target values rather than verified absolute pressures. Nevertheless, the accuracy of the iFalcon^TM^V700 tonometer series in rabbit eyes has been established in previous validation studies [24, 29]. The ex vivo rabbit eye was fixed in the compression device, and the rebound tonometer was mounted on a dedicated tonometer holder. All components - the ex vivo rabbit eye, compression device, tonometer holder, and tonometer - were secured to an adjustment platform. This enabled controlled adjustment of the measuring distance and position between the probe and the cornea.

**Fig. 2:**
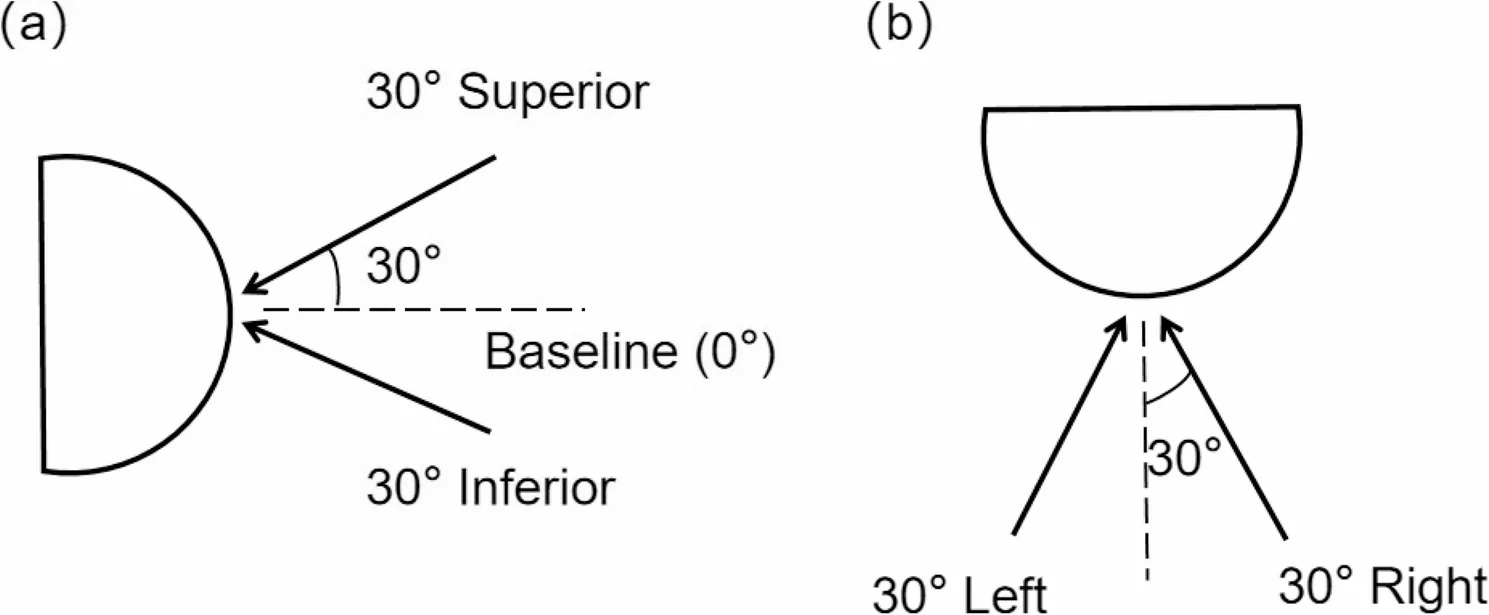
Schematic diagrams of measurement angles. Panel (**a**) represents the sagittal view, panel (**b**) represents the axial view

In the angular measurement experiment (Fig. 2), the baseline condition was defined as the probe being perpendicularly aligned with the corneal apex (0°). The same operator held the tonometer manually and tilted the probe approximately 30° relative to the central corneal normal from four directions: left, right, superior, and inferior, while keeping the probe aimed at the corneal center. The built-in gyroscope of the tonometer provided real-time angular feedback, allowing the operator to maintain the intended tilt angle within ± 3° throughout measurements. The 30° angle was chosen because it represents a clearly discernible deviation angle that is practically relevant for routine manual operation, enabling consistent reproduction across the four tested directions. All angular measurements were performed by the same operator to minimize variability. Due to manual operation, minor deviations in the actual angle may exist, and this limitation is addressed in the Discussion section.

**Fig. 3:**
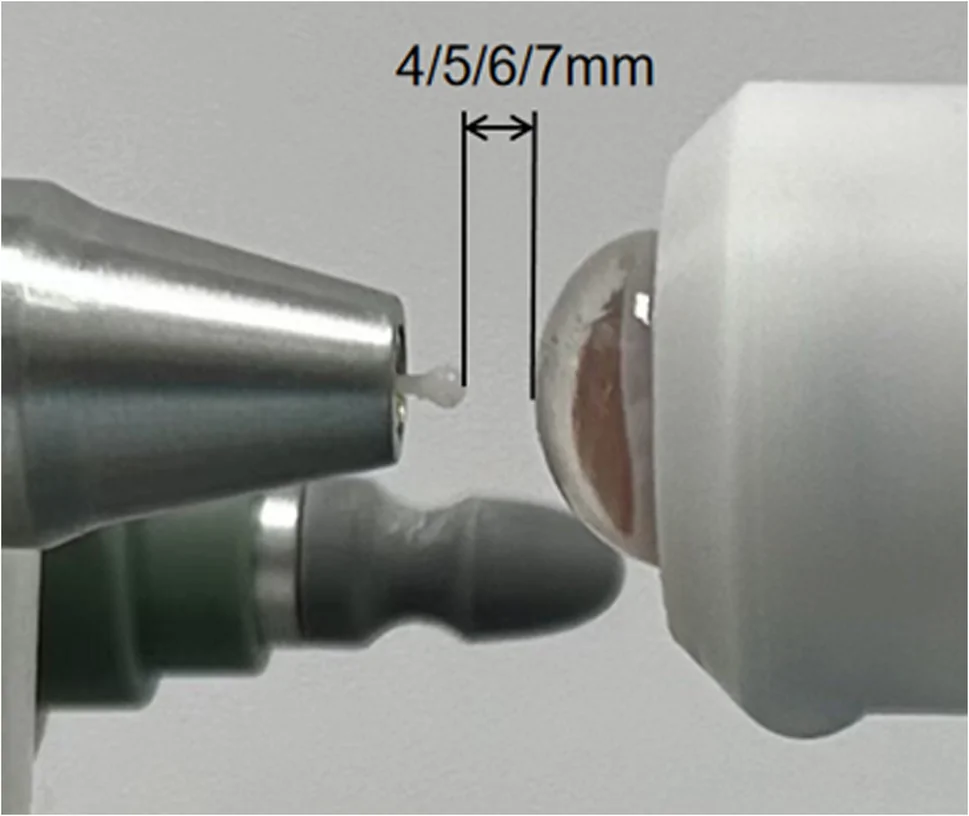
Schematic diagrams of measurement distances

**Fig. 4:**
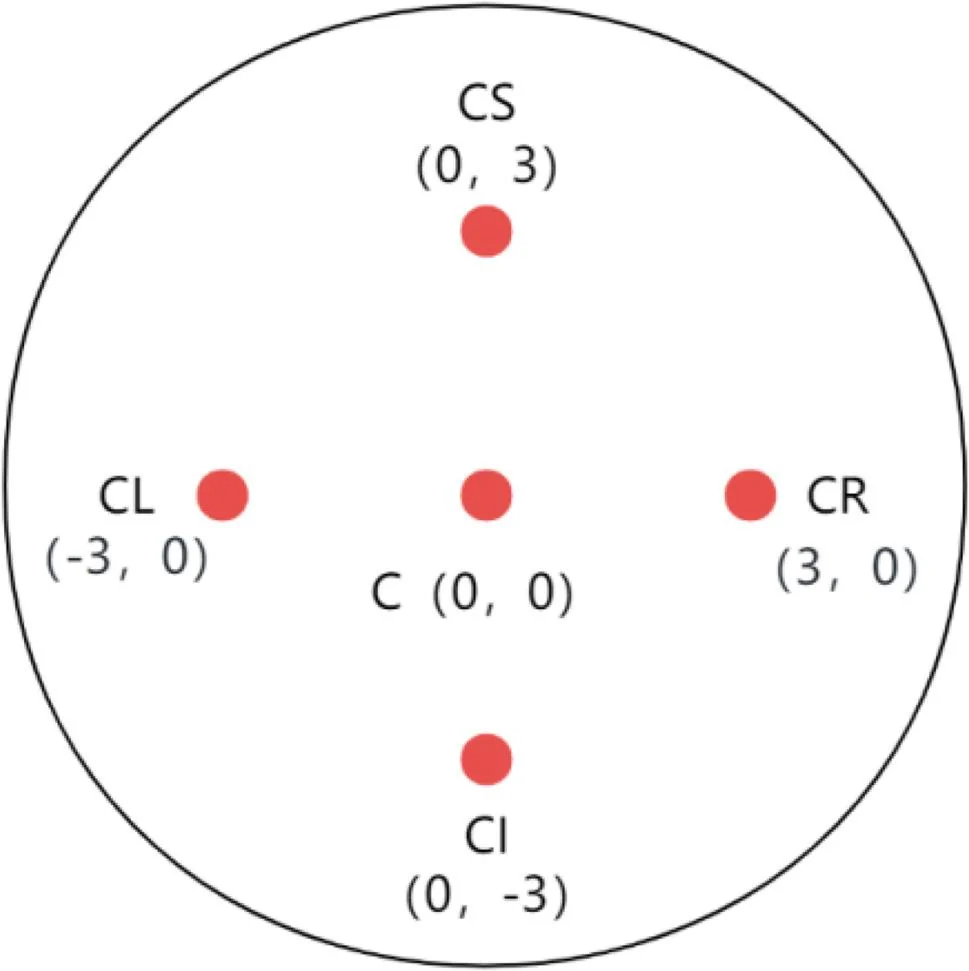
Schematic diagrams of measurement positions

For distance and position measurements, the probe-cornea relationship was manually controlled using the aforementioned adjustment platform. The operator moved the tonometer holder to the position marked on the platform scale and then fixed it for measurement. This method enables relatively accurate control of distance and position but with limited precision. Specifically, in the distance experiment (Fig. 3), the distance between the probe and corneal apex was set to 4, 5, 6, and 7 mm, respectively. In the position experiment (Fig. 4), four measuring sites were established at 3 mm left, 3 mm right, 3 mm superior, and 3 mm inferior from the corneal apex. The corneal apex was defined as the reference center (0,0). The probe remained perpendicular to the corneal surface. That is, the incidence direction was parallel to the central corneal normal. Only the impact point on the cornea was changed. All directional descriptions (superior, inferior, left, right) are based on the observer’s perspective when facing the corneal surface.

Eight IOP levels were set under different test conditions: 10, 15, 20, 25, 30, 35, 40, and 50 mmHg. The listed IOP gradients represent nominal target levels; the actual achieved pressures may deviate slightly from these values, as pressure was adjusted manually by turning the lead screw and could only be stabilized around each intended target. Measurements were performed after waiting for 10 s following adjustment to each target pressure level to allow the pressure to stabilize. Of note, no independent pressure sensor was used as the gold standard in this study. The reported biases specifically refer to the relative differences in IOP readings between different operating conditions and the baseline. They are not absolute errors between measured values and true IOP. Consequently, while the present findings characterize the consistency of the device under varying procedural conditions, they do not permit conclusions regarding its absolute accuracy. This limitation should be considered when interpreting the reported bias magnitudes and is further addressed in the Discussion section.

IOP was measured using the iFalcon^TM^V700 rebound tonometer set to the rabbit-specific operation mode. Six consecutive readings were recorded at each pressure point. The device has built-in modes for several species, including rabbit, cat, horse, dog, monkey, and rat. We selected the rabbit mode. The manufacturer indicates that this mode has been calibrated for rabbit IOP. All measurements were performed in a laboratory environment at room temperature (approximately 22 ± 2 °C); relative humidity was not controlled or recorded. A new disposable probe was used for each rabbit eye before the experiment to prevent cross-contamination.

### Statistical analysis

All statistical analyses were performed using Python 3.12. To evaluate the effects of measuring distance, angle, and position on IOP readings, paired t-tests [30–32] were used for pairwise comparisons between each operating condition and the baseline condition. In this study, two levels of pairing were applied: (1) six repeated measurements obtained under each operating condition were averaged to generate a single mean value per condition per eye per IOP level; (2) each test-condition mean was paired with the corresponding reference-condition mean obtained from the same eye at the same target IOP. This paired design effectively controlled for inter-eye variability and baseline IOP differences. The paired t-test was chosen because the paired differences were approximately normally distributed and the sample consisted of 8 independent IOP levels (df = 7), satisfying the assumptions of the test. The baseline was defined as 4 mm distance, middle angle, and central corneal position (0,0) [33]. The mean difference (MD), standard deviation (SD), standard error (SE), 95% confidence interval (95% CI), and P-value were calculated. The MD reflects the systematic deviation of each operating condition from the baseline. The 95% CI was used to assess the statistical significance and potential range of the deviation. Notably, given the limited sample size (2 rabbit eyes) in this study, the normality assumption underlying the paired t-test could not be effectively validated. Therefore, we supplemented our analysis with the Wilcoxon signed-rank test. Both tests yielded consistent statistical inferences. The core inference of this study was based on the magnitude of bias and its practical significance, as reflected by descriptive statistics, rather than strict statistical significance. The t-test are for reference only serve as supplementary tools [34, 35]. For the same reason, no adjustment for multiple comparisons was applied, and this limitation is addressed in the Discussion section. In addition, Bland-Altman plots [36–39] were constructed to assess agreement between each test condition and its corresponding reference condition. The analysis was performed using individual repeated measurements rather than averaged values, providing a more comprehensive representation of the data distribution. The limitation regarding measurement pseudoreplication has been addressed in the Discussion section.

To investigate the potential influence of IOP gradients on measurements across different operating conditions, the average value (AV), standard deviation (SD), effect sizes (Cohen’s d) and condition-between deviation (CBD) were calculated for each target pressure. Effect sizes were evaluated using Cohen’s d, with thresholds interpreted as small (d ≥ 0.20), medium (d ≥ 0.50), and large (d ≥ 0.80) according to Cohen [40]. CBD was defined as the mean absolute deviation of each condition mean from the overall mean of all condition means at that pressure level: $\:CBD=\frac{1}{n}\varSigma\:\left|{x}_{\dot{i}}-\stackrel{-}{x}\right|$, where $\:{x}_{\dot{i}}$ represents the mean value under each operating condition, $\:\stackrel{-}{x}$ is the mean of all condition means at that pressure level, and nis the number of operating conditions. Line charts were plotted using the matplotlib library. The significance level for all hypothesis tests was set at α = 0.05 for descriptive reference. However, given the limited sample size (*n* = 2 eyes), the primary interpretation of the results was based on the magnitude of the MD and their 95% CI, rather than on P-values alone. Paired t-test results are provided as supplemental information in support of the descriptive findings.

## Results

The distance, angle, and position experiments were performed as separate runs. The pressure gradients in each table represent nominal target levels, and actual achieved IOP values may vary between runs due to manual pressure adjustment. All within-table comparisons are internally valid as each condition was paired against its own reference within the same run.

### Measuring distance

**Fig. 5:**
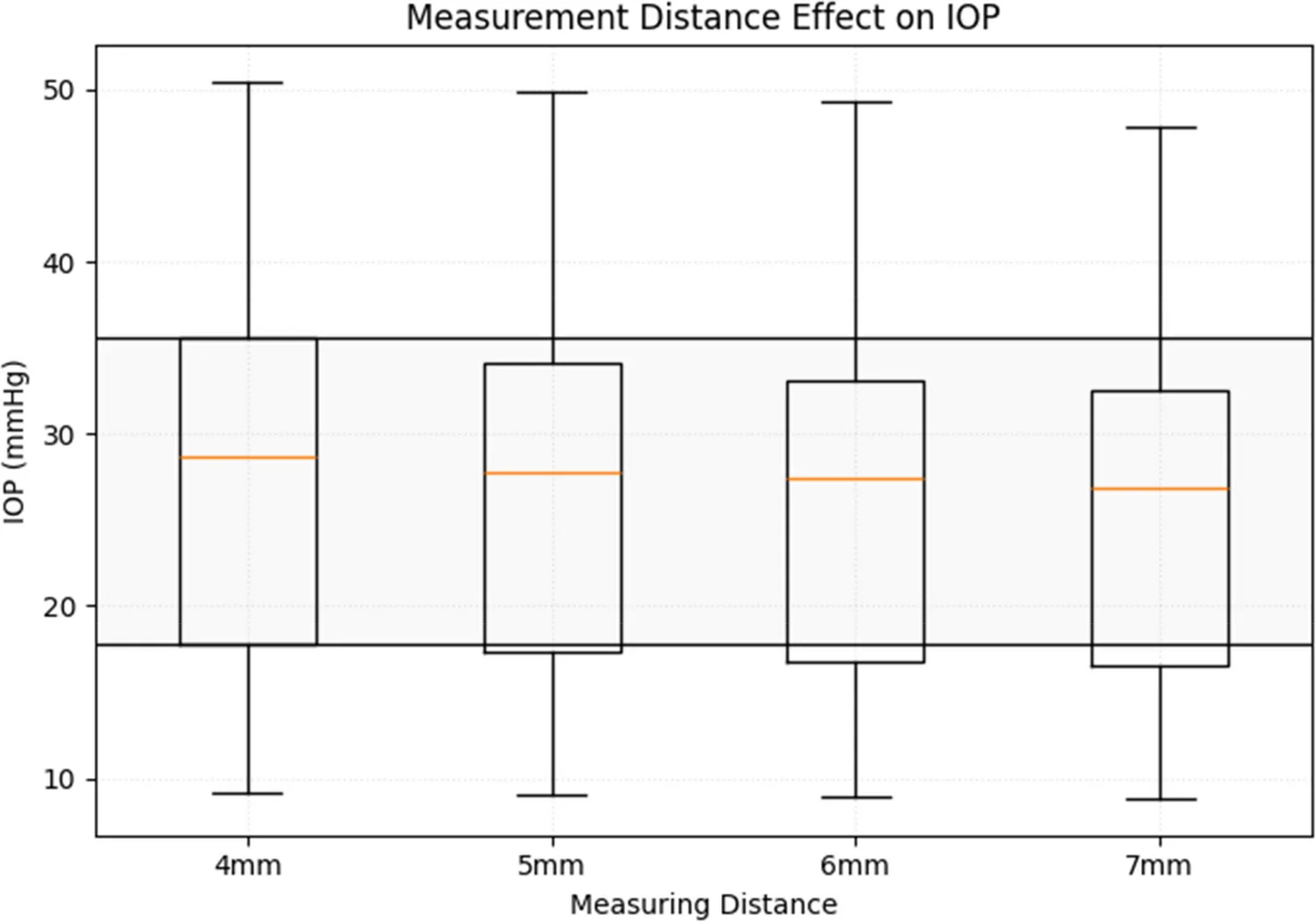
Box plot of IOP readings at different measurement distances using the iFalcon^TM^V700

Box plot analysis (Fig. 5) revealed that increasing measurement distance led to reductions in both median values and interquartile ranges, indicating that greater operating distance contributes to IOP reading underestimation.

**Fig. 6:**
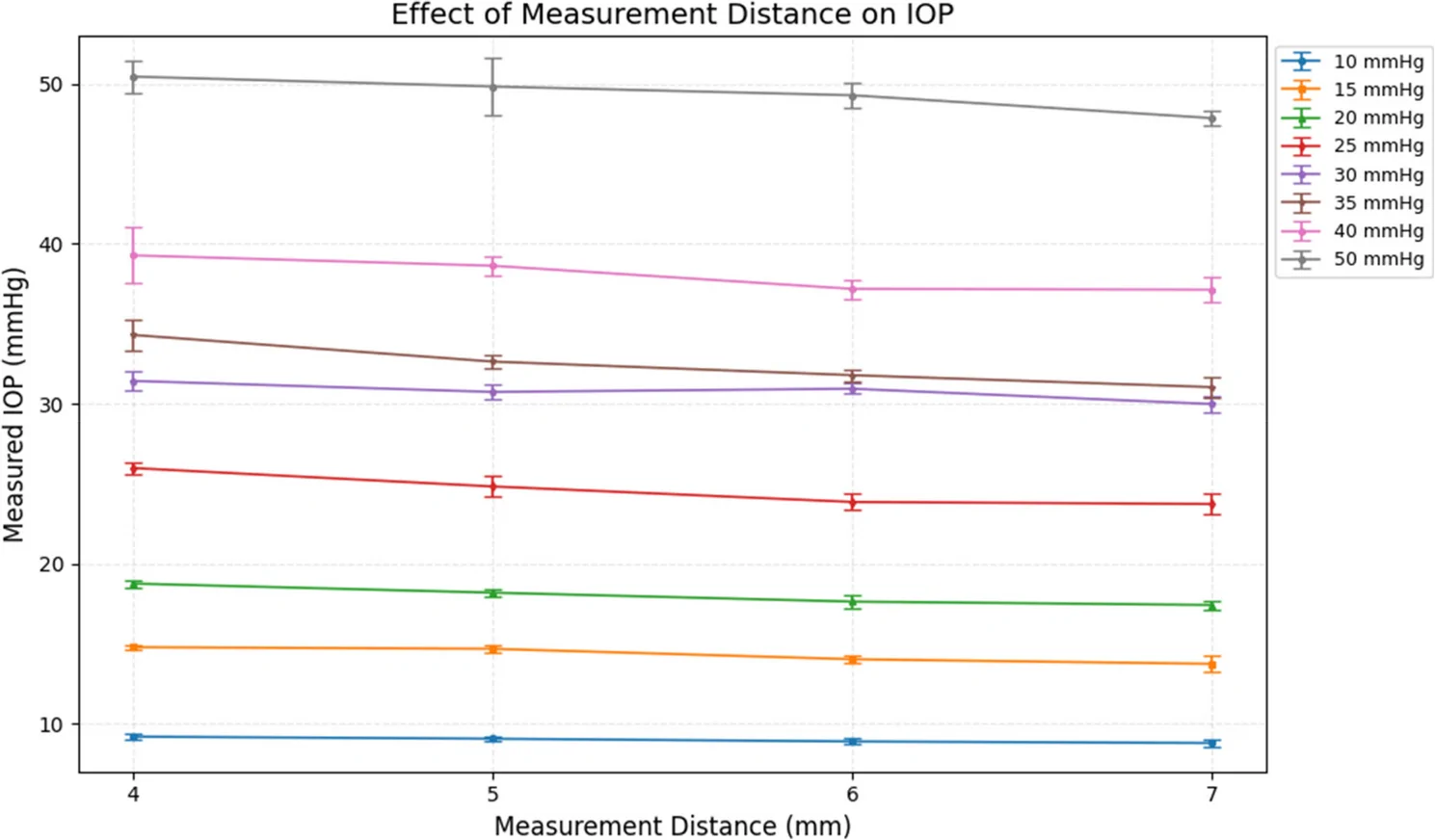
Line plot analysis of IOP readings across target IOP levels at varying measurement distances

Line chart analysis (Fig. 6) demonstrated that within the target IOP range of 10–50 mmHg, IOP readings at each pressure level exhibited a mild downward trend as measurement distance increased from 4 to 7 mm, and the magnitude of this trend increased slightly with elevated IOP, indicating that the influence of distance on readings was more pronounced at high IOP levels.

In the 10–20 mmHg IOP interval, the lines were nearly horizontal, with no visually discernible differences in IOP readings across distances. Error bars for all data points were narrow and highly overlapping, indicative of good measurement repeatability. Although the moderate-to-high IOP interval (25–50 mmHg) showed a clearer downward trend, the overall reduction was mild. Even at the maximum target IOP of 50 mmHg, readings obtained at 7 mm distance were only slightly lower than those at 4 mm.

The error bars at each measurement distance remained generally stable, with only minor fluctuations at individual points. No significant expansion of data dispersion was observed with increasing distance, indicating that such fluctuations were more likely attributable to random errors in single measurements or subtle operational variations, rather than reduced measurement stability induced by distance itself. Overall, within the practical operating distance range of 4–7 mm, IOP readings showed no obvious systematic shift alongside distance variation, and the device demonstrated favorable operational stability.

Further paired t-tests were conducted using 4 mm as the reference distance to quantify differences in IOP readings at 5 mm, 6 mm, and 7 mm (Table 1). The results revealed that readings at all tested distances were significantly lower than those at 4 mm (*p* < 0.01), and the magnitude of bias increased incrementally as distance rose. At the maximum tested distance (7 mm), MD reached − 1.8125 mmHg (95% CI: −2.5863 to − 1.0387, *P* < 0.001). The corresponding Cohen’s d values ranged from − 1.355 to -1.958, indicating large effect sizes for all distance-related comparisons, consistent with the statistically significant differences observed. Nevertheless, such absolute bias remained minor from a practical perspective. [22, 41, 42] In addition, the SD (0.5147–0.9255) and SE (all < 0.33) across all pairwise comparisons were low, indicating limited data dispersion and high measurement stability. Furthermore, the 95% CI for all distance-based comparisons were moderately wide and did not span zero, confirming the statistical significance of the observed bias [39]. However, the absolute values of the interval bounds did not exceed 2.6 mmHg, indicating that although the bias was consistently detectable, its overall magnitude remained limited.

**Table 1:** Paired comparisons of IOP readings at different measurement distances

	MD	SD	SE	t-value	*P*-value	95% CI	Cohen’s d
5 mm vs. 4 mm	-0.6975	0.5147	0.1820	-3.8332	0.0064	[-1.1278, -0.2672]	-1.3552
6 mm vs. 4 mm	-1.3225	0.8282	0.2928	-4.5167	0.0027	[-2.0149, -0.6301]	-1.5968
7 mm vs. 4 mm	-1. 8125	0.9255	0.3272	-5.5390	0.0009	[-2.5863, -1.0387]	-1.9584

**Fig. 7:**
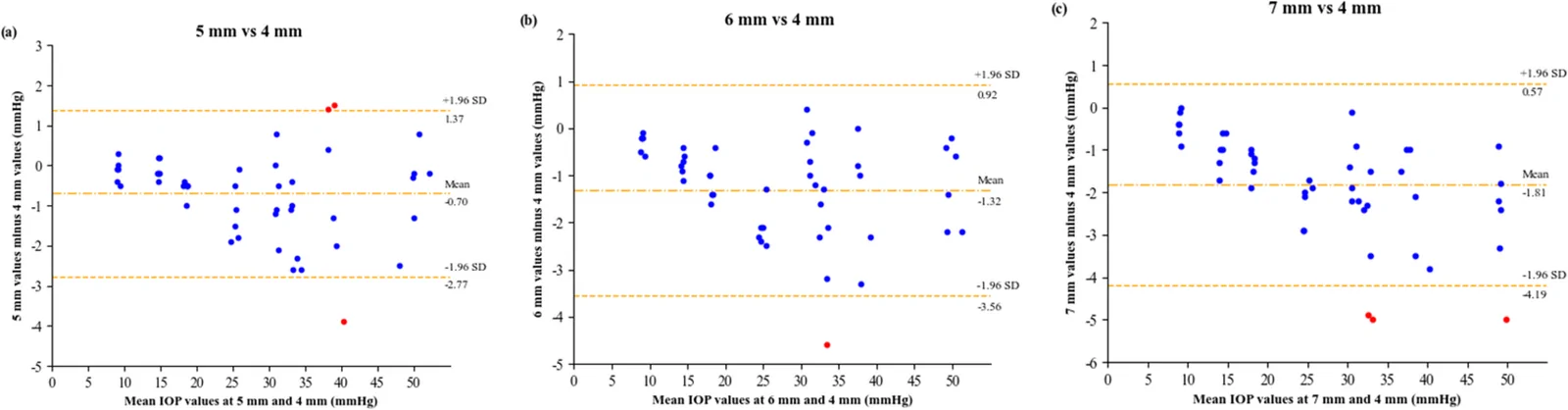
Bland-Altman plots for intraocular pressure (IOP) readings obtained at (**a**) 5 mm, (**b**) 6 mm, and (**c**) 7 mm measurement distances versus the reference distance of 4 mm using the iFalcon^TM^V700 rebound tonometer in ex vivo rabbit eyes

The Bland-Altman plots for distance comparisons (Fig. 7) revealed that the widest LoA among the three distance comparisons ranged from − 4.19 to 0.57 mmHg (7 mm vs. 4 mm), and the LoA width tended to increase with measurement distance.

Further analysis was conducted to explore the changing trend of distance effects across different IOP levels, as summarized in the corresponding table (Table 2). The SD and CBD values overall presented a rising trend as IOP elevated. In the 10–20 mmHg IOP interval, SD ranged from 0.1776 to 0.6019 mmHg and CBD from 0.1425 to 0.4750 mmHg, indicating highly concentrated IOP readings. In the moderate-to-high IOP interval (30–50 mmHg), [27, 43, 44] SD increased to 0.6026–1.4047 mmHg and CBD to 0.4125–1.0300 mmHg, with a notable increase in data dispersion [45]. Although minor fluctuations occurred at 35 mmHg, the general upward trend remained distinct, suggesting that the influence of distance on measurements intensified with increasing IOP. In brief, reading fluctuations induced by distance deviation were more prominent under high IOP conditions, consistent with the observation that the slope in the line charts increased with elevated IOP. Overall, even at high IOP levels, the maximum SD and CBD values were 1.4047 mmHg and 1.0300 mmHg, respectively. All variations remained within a narrow scope, further confirming that the tonometer maintained acceptable measurement stability at elevated pressure levels.

**Table 2:** Summary statistics of IOP readings at different measurement distance across target IOP levels

IOP	AV	SD	CBD
10 mmHg	8.9725	0.1776	0.1425
15 mmHg	14.2975	0.5037	0.4225
20 mmHg	17.9900	0.6019	0.4750
25 mmHg	24.5900	1.0427	0.8050
30 mmHg	30.7625	0.6026	0.4125
35 mmHg	32.4350	1.4047	1.0300
40 mmHg	38.0550	1.0728	0.9000
50 mmHg	49.3725	1.1098	0.7875

### Measuring angle

**Fig. 8:**
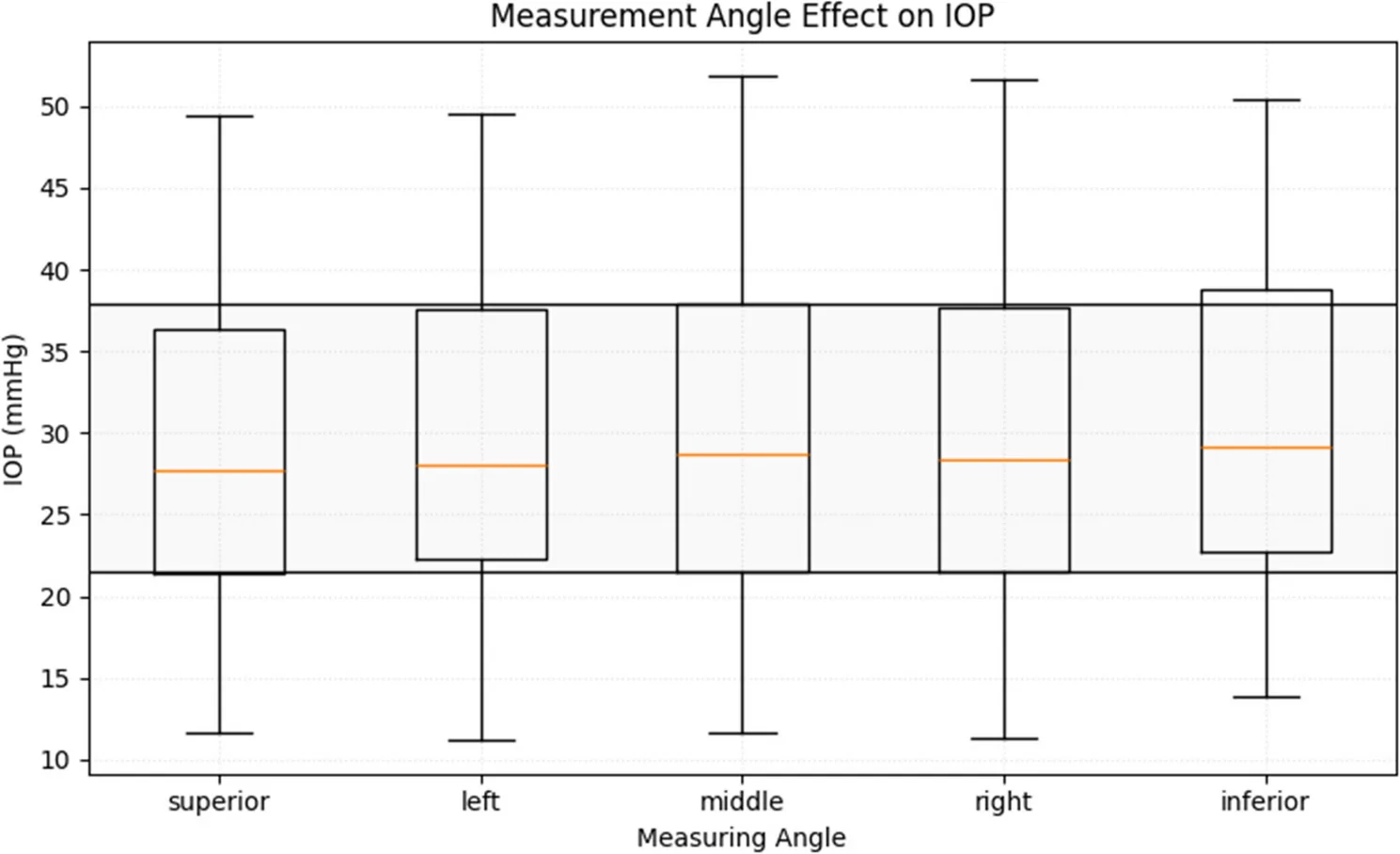
Box plot analysis of IOP readings at different measurement angles using the iFalcon^TM^V700

Box plot analysis (Fig. 8) revealed that median values and interquartile ranges were lower in the superior direction, marginally higher in the inferior direction, and comparable in the left and right orientations relative to central measurements, indicating that probe tilt angle can induce measurable differences in IOP readings.

**Fig. 9:**
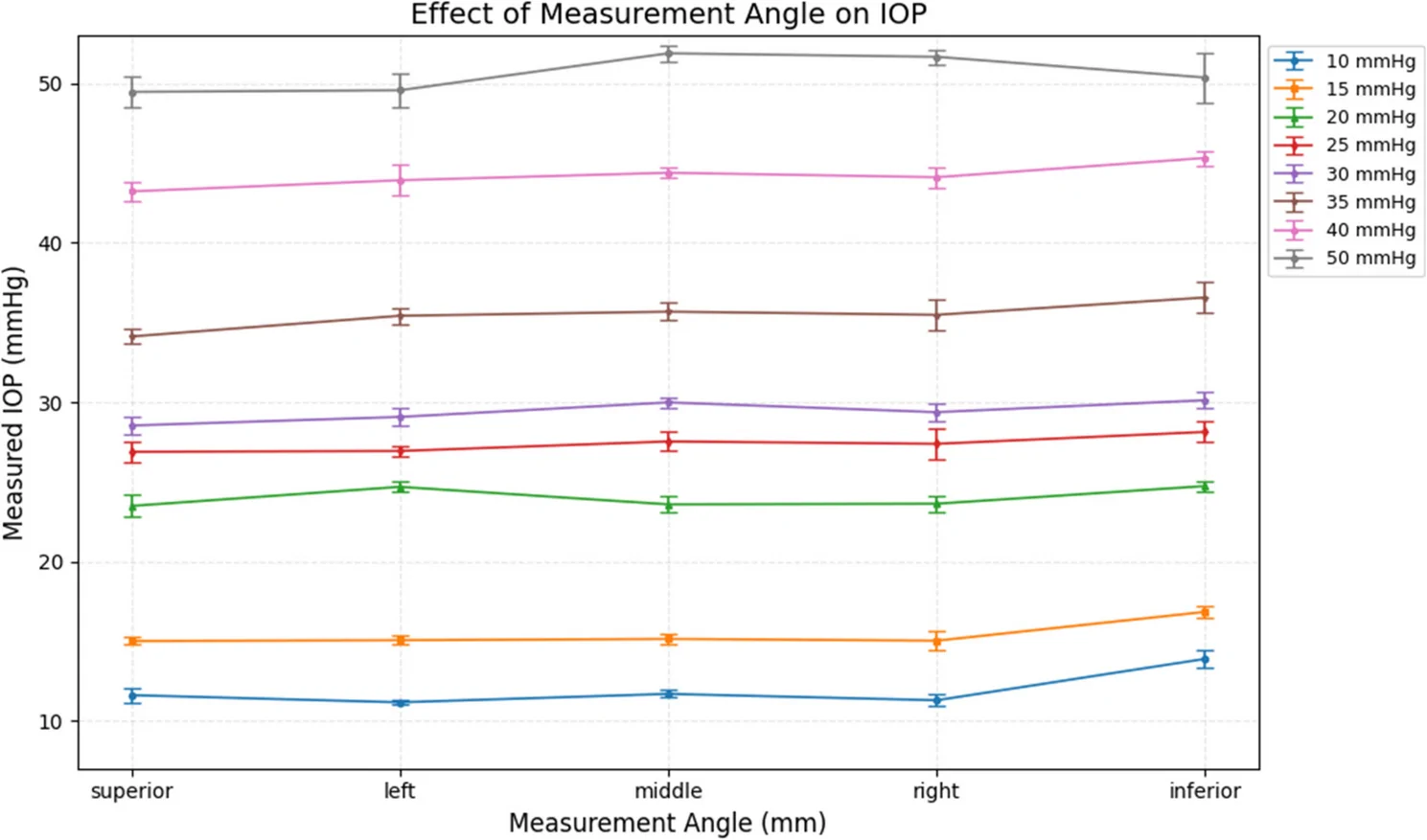
Line plot analysis of IOP readings across target IOP levels at varying measurement angles

Line graph analysis (Fig. 9) demonstrated an overall parallel distribution among the curves, indicating a generally consistent response pattern of the tonometer under different angular orientations. Among the five measuring angles, IOP readings in the inferior direction were marginally higher overall than those in other directions, with such discrepancies becoming more prominent within the moderate-to-high IOP interval (25–50 mmHg). By comparison, readings obtained in the superior direction were lower. Middle, left, and right readings remained intermediate between the two, with only minor numerical disparities across these groups.

Despite these fluctuations, curves for all angular conditions exhibited general consistent trends, with narrow error bars and high overlap, no obvious crossing or abnormal dispersion, intuitively suggesting that the device retains good tolerance to routine angular tilts.

Further paired t-tests were conducted with the middle angle as the reference to compare IOP readings across the four tilted directions: superior, inferior, left, and right (Table 3). The results revealed that MD between the superior direction and the middle direction was − 0.9450 mmHg (95% CI: −1.6567 to − 0.2333, *P* = 0.0164, cohen’s d: -1.1101), while the right direction showed an MD of − 0.2413 mmHg (95% CI: −0.4053 to − 0.0772, *P* = 0.0103, cohen’s d: -1.2299), both indicating statistical significance. MD between the inferior direction and the middle direction was + 0.7613 mmHg (95% CI: −0.1742 to 1.6967, *P* = 0.0957, cohen’s d: 0.6804), and the left direction showed a mean difference of − 0.5087 mmHg (95% CI: −1.3010 to 0.2835, *P* = 0.1727, cohen’s d: -0.5368), neither of which reached statistical significance (*P* > 0.05).

**Table 3:** Paired comparisons of IOP readings at different measurement angles

	MD	SD	SE	t-value	*P*-value	95% CI	Cohen’s d
superior vs. middle	-0.9450	0.8513	0.301	-3.1397	0.0164	[-1.6567, -0.2333]	-1.1100
left vs. middle	-0.5087	0.9476	0.3350	-1.5185	0.1727	[-1.3010, 0.2835]	-0.5368
right vs. middle	-0.2413	0.1962	0.0694	-3.4777	0.0103	[-0.4053, -0.0772]	-1.2299
inferior vs. middle	0.7613	1.1189	0.3956	1.9243	0.0957	[-0.1742, 1.6967]	0.6804

In terms of bias magnitude, even the statistically significant comparisons showed small absolute deviations: the superior direction was approximately 0.95 mmHg lower than the middle direction, and the right direction was approximately 0.24 mmHg lower. The SD (0.20–1.12 mmHg) and SE (0.07–0.40 mmHg) across all pairwise comparisons remained within an acceptable range, indicating limited data dispersion.

Notably, although the inferior direction did not show statistical significance, it exhibited a positive MD (+ 0.76 mmHg) with a relatively wide 95% CI (− 0.17 to 1.70), suggesting moderate measurement variability in this direction. However, no significant difference was detected, possibly limited by the sample size or data distribution. The Cohen’s d values for the four directions were − 1.110, − 1.230, 0.680, and − 0.537, respectively; the superior and right directions showed large effect sizes, while the inferior and left directions exhibited moderate-to-large effects, suggesting that the lack of statistical significance for the latter two may be attributable to the larger data variability (SD) in these measurement directions.

**Fig. 10:**
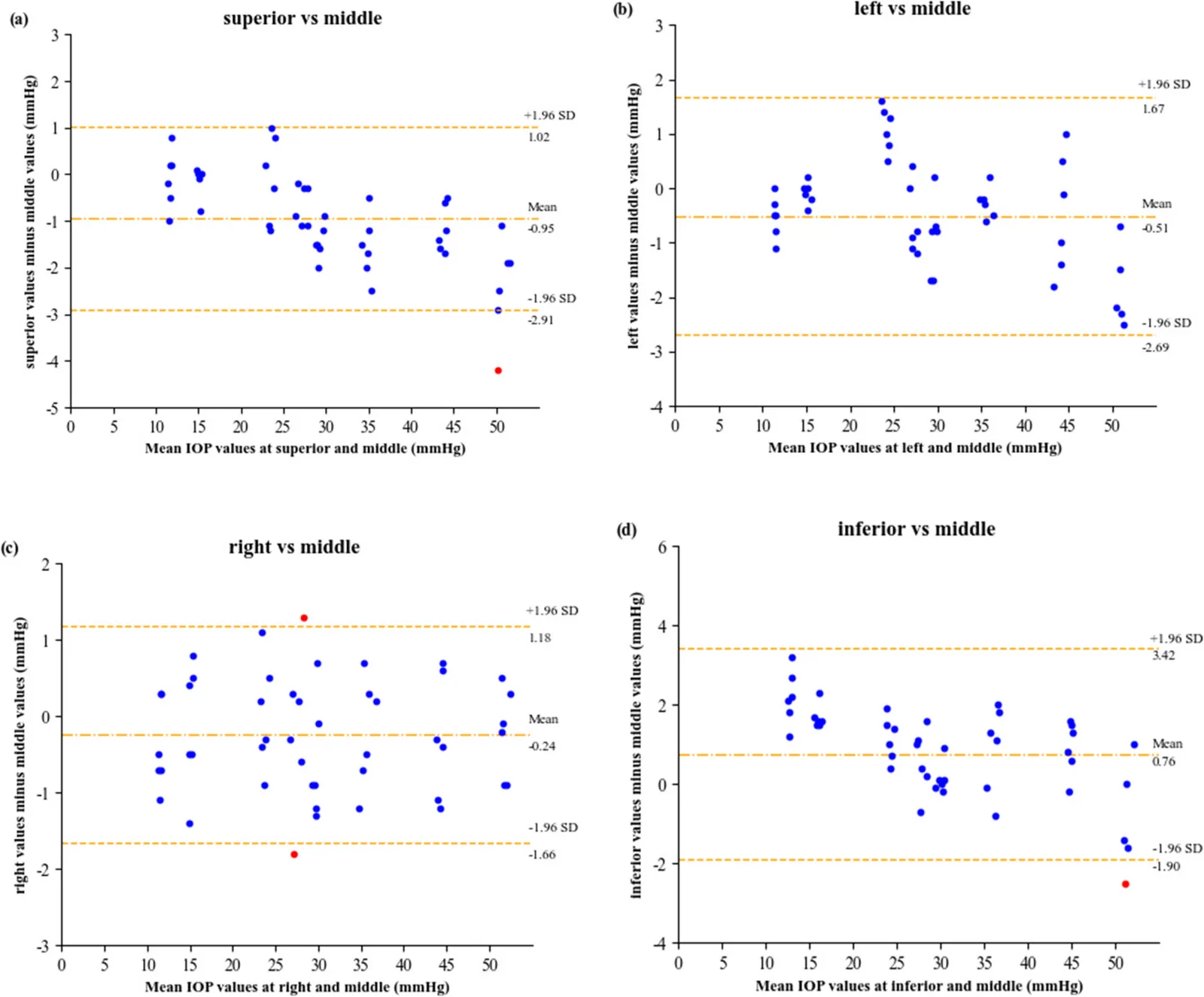
Bland-Altman plots for IOP readings obtained at four tilted measurement angles versus the reference angle (central perpendicular, middle): (**a**) superior, (**b**) left, (**c**) right, and (**d**) inferior. Each direction was tilted approximately 30° relative to the central corneal normal

The Bland-Altman plots for angular comparisons (Fig. 10) revealed that the widest LoA among the four angular comparisons ranged from − 1.90 to 3.42 mmHg (inferior vs. middle).

Further analysis was conducted to explore the trend of the angle effect across different IOP levels, as summarized in the corresponding table (Table 4). The overall SD and CBD remained at low levels, with SD ranging from 0.5092 to 1.1401 mmHg and CBD ranging from 0.3720 to 0.9488 mmHg.

**Table 4:** Summary statistics of IOP readings at different measurement angle across target IOP levels

IOP	AV	SD	CBD
10 mmHg	11.9180	1.1185	0.7848
15 mmHg	15.4060	0.7976	0.5696
20 mmHg	24.0180	0.6295	0.5496
25 mmHg	27.3700	0.5092	0.3720
30 mmHg	29.4140	0.6558	0.5088
35 mmHg	35.4540	0.8772	0.5472
40 mmHg	44.2020	0.7644	0.5304
50 mmHg	50.6040	1.1401	0.9488

Within the 10–20 mmHg IOP range, SD and CBD presented only mild fluctuations (SD = 1.1185 mmHg, CBD = 0.7848 mmHg at 10 mmHg). As IOP elevated to the 25–40 mmHg range, both indicators declined to relatively low levels (SD: 0.5092–0.8772 mmHg; CBD: 0.3720–0.5472 mmHg). At 50 mmHg, SD and CBD increased moderately once more (SD = 1.1401 mmHg, CBD = 0.9488 mmHg).

Despite these fluctuations, no distinct overall upward or downward trend was identified, and the dispersion remained low across all IOP gradients. These findings indicate that the influence of angle on measurements did not change significantly with varying IOP, and the measurement variability induced by angular tilts remained relatively stable across the full IOP range.

Overall, the maximum values of SD and CBD were 1.1401 mmHg and 0.9488 mmHg, respectively, with overall variation well controlled within a narrow range, indicating that the tonometer exhibits good tolerance to angular tilts at all IOP levels.

### Measuring position

**Fig. 11:**
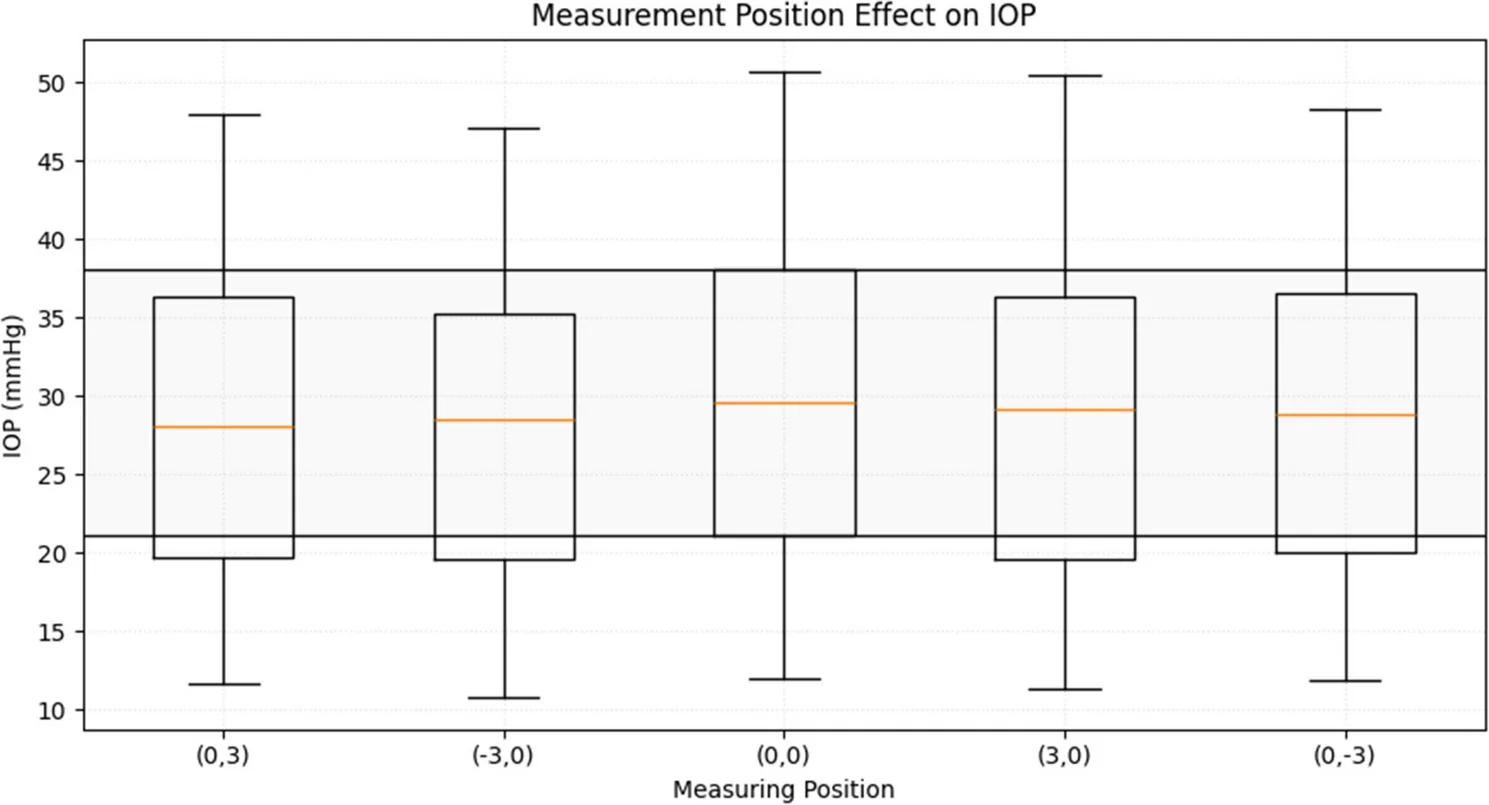
Box plot analysis of IOP readings at different corneal measurement positions using iFalcon^TM^V700

Box plot analysis (Fig. 11) revealed that the median and interquartile range at peripheral corneal positions were lower than those at (0,0), suggesting that non-central measurement locations may trigger mild underestimation of the readings.

**Fig. 12:**
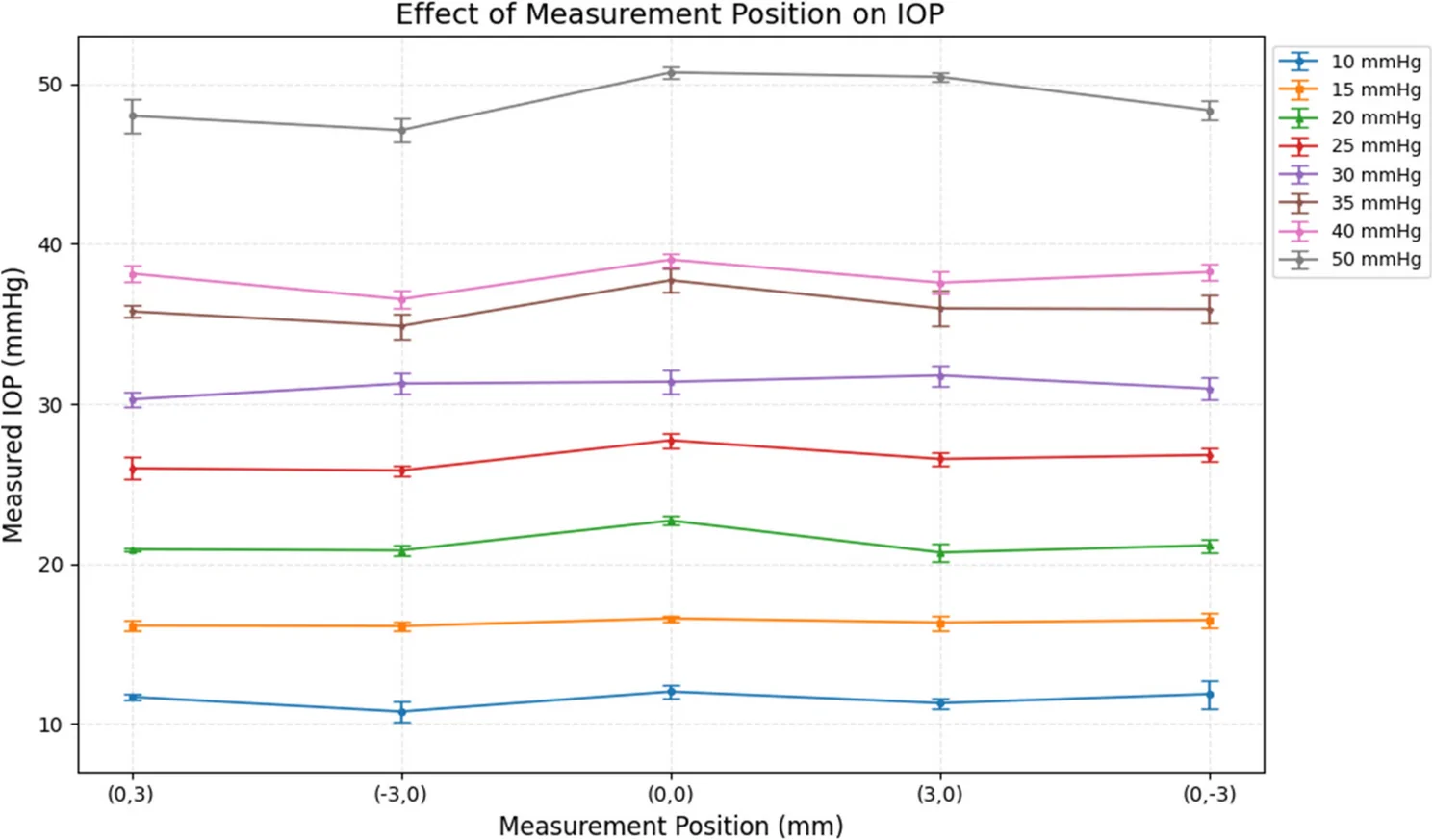
Line plot analysis of IOP readings across target IOP levels at different corneal measurement positions

Line chart analysis (Fig. 12) demonstrated that IOP readings across different corneal sites presented an overall symmetric distribution with the corneal center (0,0) serving as the peak value. Central corneal readings were the highest, whereas those at peripheral positions declined to varying degrees, forming a general pattern of central elevation and peripheral reduction.

Among the peripheral detection positions, readings at (0, − 3) were slightly higher than at (0, 3), and readings at (3, 0) were slightly elevated relative to those at (− 3, 0). Nevertheless, the overall magnitude of numerical discrepancies across all positions remained minor. All error bars remained within narrow ranges with no obvious erratic fluctuations, indicating favorable measurement repeatability. These subtle deviations between the central and peripheral regions, as well as among different quadrants, may be attributed to the non-spherical geometry of the ex vivo rabbit globes and uneven distribution of posterior supporting tissues. It is possible that such uneven distribution leads to slight differences in biomechanical transmission across orientations during flat-plate compression, which could in turn affect rebound signal acquisition. However, this interpretation remains speculative, as the present study did not directly measure corneal geometry or posterior tissue support.

Overall, within the central and 3 mm peripheral corneal range, IOP readings showed no obvious systematic shift following positional variation, and this tonometer demonstrated favorable tolerance to positional deviation.

Further paired t-tests (Table 5) were conducted using the central corneal site (0,0) as the reference to compare IOP readings at the four peripheral positions: (0, 3), (3, 0), (0, − 3), and (− 3, 0). The results revealed that IOP readings at all peripheral positions were significantly lower than central corneal values (all *P* < 0.05), with overall bias magnitudes ranging from − 0.9037 to − 1.8175 mmHg.

**Table 5:** Paired comparisons of IOP readings at different measurement positions

	MD	SD	SE	t-value	*P*-value	95% CI	Cohen’s d
(0,3) vs. (0,0)	-1.3700	0.8205	0.2901	-4.7228	0.0022	[-2.0559, -0.6841]	-1.6697
(3,0) vs. (0,0)	-0.9037	0.8307	0.2937	-3.0772	0.0179	[-1.5982, -0.2093]	-1.0879
(0,-3) vs. (0,0)	-1.0112	0.8225	0.2908	-3.4777	0.0103	[-1.6988, -0.3237]	-1.2294
(-3,0) vs. (0,0)	-1.8175	1.1808	0.4175	-4.3535	0.0033	[-2.8047, -0.8303]	-1.5392

Among all peripheral groups, the location in the (− 3, 0) direction was the largest, with an MD of − 1.8175 mmHg (95% CI: −2.8047 to − 0.8303, *P* = 0.0033). SD values of each group ranged from 0.8205 to 1.1808, and the SE ranged from 0.2901 to 0.4175, remaining at low levels overall, indicating low dispersion and good stability of the data.

None of the 95% CI spanned zero, and all Cohen’s d values exceeded 0.80 in absolute magnitude, further confirming a genuine systematic positional bias effect. The maximum absolute value of the upper and lower limits was 2.8047 mmHg, suggesting that although stable deviations exist between the central and peripheral corneal measuring points, the overall bias magnitude is limited and its impact on practical tonometry outcomes remained limited.

**Fig. 13:**
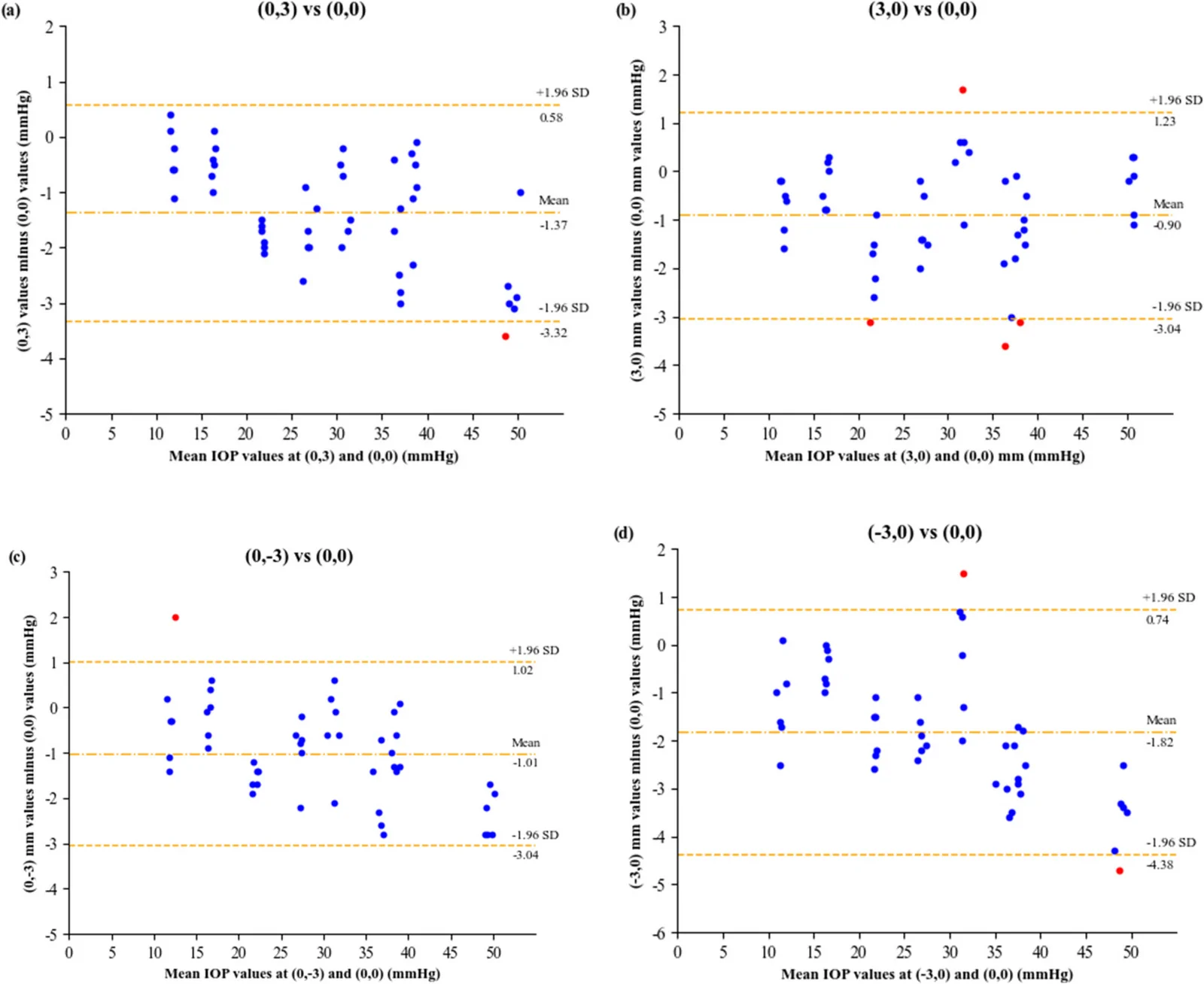
Bland-Altman plots for IOP readings obtained at four peripheral corneal measurement positions versus the central corneal reference position (0,0): (**a**) (0, 3) mm, (**b**) (3, 0) mm, (**c**) (0, − 3) mm, and (**d**) (− 3, 0) mm

The Bland-Altman plots for positional comparisons (Fig. 13) revealed that the widest LoA among the four positional comparisons ranged from − 4.38 to 0.74 mmHg ((− 3, 0) vs. (0, 0)).

Further analysis was conducted to explore the trend of the position effect across different IOP levels, as summarized in the corresponding table (Table 6). SD varied from 0.2108 to 1.5795 mmHg, while CBD ranged from 0.1664 to 1.3232 mmHg. Notably, in the 10–15 mmHg range, both SD and CBD remained at low levels (SD ≤ 0.50 mmHg, CBD ≤ 0.40 mmHg). In the 20–40 mmHg interval, SD and CBD exhibited mild fluctuations yet maintained favorable overall stability (SD: approximately 0.5611–1.0378 mmHg, CBD: 0.4136–0.6760 mmHg). At 50 mmHg, data dispersion increased markedly (SD = 1.5795 mmHg, CBD = 1.3232 mmHg), representing the highest values throughout the tested range.

**Table 6:** Summary statistics of IOP readings at different corneal measurement positions across target IOP levels

IOP	AV	SD	CBD
10 mmHg	11.5100	0.5029	0.3960
15 mmHg	16.3220	0.2108	0.1664
20 mmHg	21.2560	0.8237	0.5776
25 mmHg	26.5740	0.7555	0.5488
30 mmHg	31.1320	0.5611	0.4136
35 mmHg	36.0560	1.0378	0.6696
40 mmHg	37.9100	0.9170	0.6760
50 mmHg	48.9360	1.5795	1.3232

These findings indicate that measurement variability triggered by positional deviation is amplified under extremely elevated IOP conditions, but remains stable within the clinically prevalent range of 10–40 mmHg. Overall, data dispersion across all IOP gradients was low except at 50 mmHg, further indicating that the device exhibits good tolerance to positional deviation.

## Discussions

Rebound tonometry is widely employed for IOP monitoring in animal experiments owing to its non-invasive, rapid, and convenient characteristics. Nevertheless, its measurements are susceptible to influences from factors including operating distance, probe angle, and measurement position. Given that corneal biomechanical properties vary across species [46, 47], and discrepancies in device algorithms and detection principles, existing studies have not reached a consensus regarding the tolerance range for operating conditions. Accordingly, the present study systematically assessed the effects of measurement distance, angle, and corneal measurement location on readings obtained with the iFalcon^TM^V700 rebound tonometer in an ex vivo rabbit eye model, aiming to determine the measurement stability of this device under routine operational deviations. Experimental results show that the device demonstrates reasonable measurement stability under the tested conditions: within the intraocular pressure range of 10–50 mmHg, the maximum mean deviation (MD) caused by any single operational factor is less than 2 mmHg—a threshold generally considered acceptable in clinical and translational research [41, 42, 48, 49]. This performance is comparable to previously validated data for similar rebound tonometers in ex vivo models, with deviation magnitudes being similar or smaller. The Bland-Altman plots further revealed that, while the mean biases across all comparisons remained within ± 2 mmHg, the LoA were relatively wide under certain conditions. This pattern indicates that although the systematic bias is clinically acceptable, single measurements may carry considerable variability. Therefore, averaging multiple readings is recommended to improve measurement reliability.

It should be noted that the IOP AV reported in different tables were obtained from separate experimental runs, with manual pressure adjustment leading to slight variations in actual achieved pressures across runs. As clarified in the Results section, the listed pressure gradients represent nominal target levels, and all within-table comparisons were paired within the same run. Therefore, these between-run variations do not compromise the validity of the reported biases.

Regarding measurement distance, the present study observed that readings exhibited a mild linear downward trend with distance increasing, and this effect was more prominent in the high IOP range. The largest deviation was observed in the comparison between 7 mm and 4 mm, yielding an MD of − 1.8125 mmHg. Previous investigations utilizing ex vivo animal eyes have demonstrated that rebound tonometry exhibits satisfactory linear correlation over a measurement distance of 3–5 mm, suggesting the presence of a relatively stable distance tolerance window [15]. Although linearity was not directly assessed in the present study, analysis of measurement consistency revealed that the CBD among readings acquired at distances ranging from 4 to 7 mm remained below 2 mmHg. This observation indicates that the device maintains acceptable measurement stability over a modestly extended distance interval. Furthermore, the distance-associated bias was found to be slightly amplified at elevated IOP levels. Relative to previous reports, the magnitude of distance-related deviation in this study was smaller, suggesting that this device is less susceptible to variations in measurement distance. Inter-species differences in corneal thickness, elasticity, and curvature are likely the primary causes for numerical discrepancies [46, 47], and also indicate that the distance tolerance interval is highly species-specific and should not be directly extrapolated across different animal models.

With respect to measurement angle, this study found that angular tilts within 30° produced minimal deviations, with the maximum difference (superior vs. central) being − 0.9450 mmHg, and deviations in all directions were within ± 1 mmHg. Previous studies have reported inconsistent findings regarding angular deviation: chicken eye studies demonstrated poor tolerance of the tonometer to angular tolerance within the 0°–20° off-axis range (*P* < 0.05) [15]; canine eye studies showed that tilted or peripheral measurements resulted in only mild underestimation (differences ranging from 0 to 2.9 mmHg compared to central perpendicular measurements) [17]; in rabbit eyes, no significant alterations in IOP readings were observed within a 20° tilt angle of the probe [18]; whereas in human eyes, probe tilts of 10° or 20° caused significantly lower readings compared to Goldmann applanation tonometry [19]. Findings from the present study are aligned more closely with those reported in rabbit eyes, suggesting that angular sensitivity depends on both species-specific corneal biomechanical properties and device-specific algorithms. The more uniform corneal curvature and mechanical response of rabbit eyes compared to human and large animal models may represent an important factor underlying the small angular-related deviations observed in this study, further indicating that angular tolerance thresholds need to be established independently for each target animal model. Notably, asymmetric measurement deviations were observed between the superior and inferior directions. This directional asymmetry may be attributable to a combination of factors, including probe tilt angle, impact geometry, and gravitational orientation, each of which can affect rebound dynamics and signal acquisition [10, 50]. The left–right asymmetry was small (less than 0.3 mmHg between the left and right directions) and is most likely attributable to the intrinsic measurement variability of the rebound tonometer, with a possible minor contribution from manual manipulation. This minor asymmetry is unlikely to affect the overall interpretation of the angular results. However, the exact mechanism underlying this asymmetry remains unclear and warrants further investigation.

Regarding measurement position, the present study observed that readings at all peripheral corneal locations were significantly lower than those at the central cornea, exhibiting a symmetric pattern characterized by higher central readings and lower peripheral readings. The (-3,0) direction showed the largest deviation (MD − 1.8175 mmHg), whereas the (3,0) position showed the smallest deviation (MD − 0.9037 mmHg). Within the conventional IOP range of 10–40 mmHg, no monotonic interaction was observed between positional effect and IOP level; measurement variability remained stable with no discernible trend. However, under severely elevated IOP (50 mmHg) conditions, both the SD and CBD increased significantly, suggesting that the measurement robustness of the device diminishes at markedly elevated pressure levels. Relative to previous reports of peripheral corneal underestimation of 2–3 mmHg in canine eyes [17] and eccentric measurement deviations of 0.9–6.5 mmHg [22], the position-dependent deviation of the iFalcon^TM^V700 in this study was smaller, remaining generally within 2 mmHg. Furthermore, the SD and SE across all comparisons were low, indicative of good measurement repeatability. Potential explanations for these differences include: first, this device employs a nonlinear model based on Hertzian contact theory, which may potentially offer improved adaptability to variations in corneal curvature and regional differences in mechanical properties; second, rabbit eyes have markedly different corneal thickness, elasticity, and curvature distributions compared to large animals such as dogs, with a more uniform mechanical response; [46, 47] third, this study employed an ex vivo fixed model, eliminating confounding factors such as eye movement, blinking, and tear film fluctuations present in living animals, further enhancing measurement stability. Additionally, the non-ideal spherical structure of rabbit eyes and variations in pressure transmission caused by asymmetric posterior tissues may account for the subtle numerical differences observed between different positions [51]. 

Collectively, the iFalcon^TM^V700 rebound tonometer exhibited favorable measurement stability over a measurement distance of 4–7 mm, across various angular tilts, and within a 3-mm peripheral corneal zone, with a maximal operation-related deviation of less than 1.82 mmHg. Nevertheless, slight variations in device performance were observed under different operating conditions: the distance effect intensified with increasing IOP, leading to more pronounced reading fluctuations at elevated pressure levels; the angular effect remained relatively stable across the entire IOP range, with small deviation magnitudes in all directions; and the positional effect increased with rising IOP, particularly showing markedly greater dispersion at severely high IOP (50 mmHg), while maintaining favorable stability within the clinically conventional IOP range of 10–40 mmHg [51]. These findings underscore the importance of accounting for potential influences of operational factors when interpreting rebound tonometry data, especially in experimental designs involving diverse operating protocols. In practical terms, the device can be reliably used in routine ex vivo rabbit eye experiments at distances of 4–7 mm, along the horizontal midline, and at central or paracentral corneal locations. For high-precision studies, it is advisable to standardize the measurement distance to 4–5 mm, maintain a horizontal probe orientation, and target the central cornea to minimize deviations. During precise quantification of low IOP or monitoring of pathological ocular models, the potential impact of systematic bias should be considered, and data reliability can be improved by implementing standardized operating procedures.

### Limitations

Several limitations of the present study should be acknowledged. First, all experiments were conducted using only two ex vivo rabbit globes, resulting in a restricted sample size. While this sample size is consistent with the design of the majority of ex vivo device validation investigations in ophthalmology, it nonetheless imposes certain constraints on the generalizability of the findings. With only two biological replicates, we could not account for inter-individual variability in corneal biomechanical properties, such as central corneal thickness, hydration status, and age-related differences, all of which may influence tonometer performance [51, 52]. In addition, while repeated measurements from the same eye under identical conditions improve the precision of the estimates, they do not constitute independent biological replicates and thus do not increase the effective sample size for inference, and this limitation should be considered when interpreting the statistical results.

Second, the ex vivo experimental design carries inherent constraints. Following enucleation, corneal biomechanical properties, tissue elasticity, and other characteristics undergo post-mortem changes over time. Furthermore, the ex vivo setting cannot reproduce physiological mechanisms present in vivo, such as dynamic tear film formation and homeostatic regulation of corneal hydration [53]. Such factors may contribute to an amplification of measurement error, a limitation inherent to all ex vivo ophthalmic investigations, given that the isolated environment cannot fully replicate the in vivo ocular microenvironment.

This study was designed to investigate relative differences between operating conditions (distance, angle, and position) rather than to measure absolute IOP values. All measurements were performed in rapid succession for each condition, thereby limiting the time available for post-mortem corneal changes to develop. Consequently, our comparative findings are unlikely to be substantially confounded by ex vivo tissue alterations [54]. 

Third, certain methodological limitations warrant mention: key corneal biomechanical parameters, including central corneal thickness (CCT) and corneal hydration status, were neither measured nor controlled in the present investigation. Although slit-lamp examination ruled out obvious structural abnormalities in the eyeballs, individual differences in these uncontrolled variables may still contribute to measurement error, limiting a comprehensive analysis of sources of variation in device performance.

Fourth, the present investigation was restricted to the evaluation of a single angular deviation (approximately 30°), a measurement distance ranging from 4 to 7 mm, and a limited number of corneal locations. Systematic exploration of smaller angular increments, a wider range of measuring distances, and more corneal locations was not performed. Although the built-in gyroscope provided real-time feedback to help maintain the intended angle, manual angular adjustments may still have introduced minor variability. In addition, minor irregularities in pressure transmission from the compression apparatus may have slightly affected the measurements.

Fifth, an independent pressure transducer was not employed for verification of true IOP. Thus, the present work assessed the relative measurement consistency of the iFalcon^TM^V700 across varying procedural conditions, rather than its absolute accuracy. Readers should therefore interpret the reported bias magnitudes as relative differences between operating conditions, rather than as absolute errors against a true reference standard.

Sixth, no adjustment for multiple comparisons was applied to the paired t-tests. Although this study was exploratory in nature and emphasized bias magnitudes rather than P-values alone, the absence of correction may increase the risk of type I error (false-positive findings) for individual comparisons.

In light of the limitations outlined above, future investigations should aim to expand sample size, incorporate in vivo validation in animal models, and include finer operational gradients as well as relevant corneal biomechanical parameters. This will further improve the application evaluation system of the iFalcon^TM^V700 rebound tonometer and provide more robust evidence for its standardized use in clinical and experimental settings.

## Conclusions

This investigation was conducted using an ex vivo rabbit ocular model with a restricted sample size (*n* = 2), and an independent pressure transducer was not employed as a reference standard. Consequently, the findings represent an initial characterization of device performance under controlled conditions, rather than a definitive assessment of its clinical performance, and should not be directly extrapolated to in vivo settings or to other species.

Within the limits of this ex vivo model, the iFalcon^TM^V700 rebound tonometer was found to yield stable IOP measurements under different operating conditions. Although mild systematic biases were observed, the maximum MD induced by each operating factor was less than 2 mmHg, suggesting that these operational factors are unlikely to compromise the interpretation of experimental IOP data in rabbit studies. However, the relatively wide limits of agreement observed under certain test conditions suggest that averaging multiple readings is advisable in practical applications.

## Data Availability

The data supporting the findings of this study are available from the corresponding author upon reasonable request.

## Abbreviations

3D: Three-dimensional
AV: Average value
CBD: Condition-between deviation
CCT: Central corneal thickness
CI: Confidence interval
IOP: Intraocular pressure
CI: Confidence interval
LoA: Limits of agreement
MD: Mean difference
SD: Standard deviation
SE: Standard error
